# Wheat and Barley: Acclimatization to Abiotic and Biotic Stress

**DOI:** 10.3390/ijms21197423

**Published:** 2020-10-08

**Authors:** Tomasz Hura

**Affiliations:** Polish Academy of Sciences, The Franciszek Górski Institute of Plant Physiology, Niezapominajek 21, 30-239 Kraków, Poland; t.hura@ifr-pan.edu.pl

**Keywords:** barley, wheat, abiotic stress (drought, heat, salinity, cold, UV radiation, flooding), biotic stress (bacteria, viruses, fungi, parasites, insects, weeds), multi-stress, genes and proteins, transcriptome, proteome, metabolome

## Abstract

Twelve articles (ten research papers and two reviews) included in the Special Issue entitled “Wheat and Barley: Acclimatization to Abiotic and Biotic Stress” are summed up here to present the latest research on the molecular background of adaptation to environmental stresses in two cereal species. Crucial research results were presented and discussed, as they may be of importance in breeding aimed at increasing wheat and barley tolerance to abiotic and biotic stresses.

## 1. Introduction

I have the pleasure of introducing this Special Issue focusing on wheat and barley acclimatization to abiotic and biotic stress. Published research and review papers presented here concentrate mainly on the molecular background of wheat and barley acclimatization to adverse environmental conditions. This subject has been purposefully selected, as it concerns cereals of significant economic importance, and is also indirectly related to climate changes and increasingly often observed extreme weather events. Prolonged droughts, snowless winters or uncontrolled disease transmissions represent challenges faced by the plant production industry.

This selection of papers covers a wide range of research subjects, including both biotic (pathogen fungi, grain aphid) and abiotic (soil drought, temperature stresses, heavy metal contamination) stresses. A common thread in all these works is the determination of crucial mechanisms that operate at different levels of plant organization to improve wheat and barley tolerance to the abovementioned environmental stresses. 

The issue contains twelve interesting papers (Table 1). Similar problems associated with wheat and barley acclimatization to stressful conditions are presented from different perspectives, depending on research tools or experimental settings used, and they serve as a source of new data expanding the reader’s knowledge.

## 2. Wheat 

The research conducted by Zhang et al. [1] shows the evolution and diversification of the HIPPs (heavy metal-associated isoprenylated plant proteins) gene family in *Triticeae* species on a genome-wide scale. It also confirms that *HIPP1-V*, one of the *HIPP* genes, positively regulates tolerance in common wheat. Therefore, the authors suggest that *HIPP1-V* could be used for improving wheat resistance to Cd stress.

Liu et al. [2] presents transcriptomic changes in wheat in response to powdery mildew caused by *Blumeria graminis* f. sp. *tritici* in a continuous time period. Their results confirmed involvement of CMPG1-V, encoding the U-box E3 ubiquitin ligase, in enhancement of the broad-spectrum resistance to powdery mildew. The authors proposed a CMPG1-V-mediated molecular mechanism for the resistance to powdery mildew. Within that mechanism, they indicated signal pathways and metabolic processes that can affect the expression of pathogenesis-related genes.

Another published work focused on drought stress tolerance and photosynthetic activity of alloplasmic lines, *Triticum dicoccum x Triticum aestivum* [4]. The authors pointed out that the tetraploid species *T. dicoccum* is recognized as a potential source of drought tolerance in wheat. Their analyses demonstrated introgression of the *Dreb-B1* allele from *T. dicoccum* that was also accompanied by a high-level drought tolerance.

The article by Zhi et al. [5] describes the function of wheat histone deacetylase TaHDT701, a type of histone deacetylase 2 (HD2), in regulating the defense responses to *Blumeria graminis f. sp. tritici.*

The authors showed that TaHDT701 formed a histone deacetylase complex with RPD3-type histone deacetylase TaHDA6 and WD40-repeat protein TaHOS15. Furthermore, the analysis demonstrated that the TaHDT701–TaHDA6–TaHOS15 complex negatively regulated wheat defense responses to powdery mildew by modulating the chromatin state of defense-related genes. It is worth noticing that this is the first paper reporting the participation of HD2–RPD3–WD40 histone deacetylase complexes in the regulation of plant defense responses.

The study by Ye et al. [8] is the first report investigating two *Bipolaris sorokiniana* strains, endophytic and pathogenic, in wheat, using RNA-seq and metabolome analysis to reveal pathogenicity-related genes. *B. sorokiniana* from wheat leaf exhibited stronger pathogenicity toward wheat than endophytic *B. sorokiniana* from *Pogostemon cablin*. Measurements confirmed that pathogenicity-related genes, such as a gene encoding the loss-of-pathogenicity B (LopB) protein, cell wall-degrading enzymes, and killer and Ptr necrosis toxin-producing related unigenes, are crucial for the pathogenicity of *B. sorokiniana* from wheat leaf toward wheat. The results of these studies can significantly contribute to explaining the molecular background of *B. sorokiniana* pathogenicity. 

Czyczyło-Mysza et al. [9] present results on genetic markers associated with phenolic compounds and yield-related traits in doubled haploid lines in wheat. The authors detected 21 QTLs (for the plural: quantitative trait loci) for phenolic compounds on chromosomes 1A, 1B, 2A, 2B, 2D, 3A, 3B, 3D, 4A and 4D. Furthermore, three QTLs for both phenolics content and yield-related traits were located on chromosomes 2A, 2D and 3A. The results led to identification of some candidate genes associated with phenolics metabolism, photosynthetic pigments, yielding and photosynthetic apparatus activity that could be helpful in further research on drought tolerance in wheat. 

## 3. Barley 

In the part of the Special Issue on barley, Saja et al. [3] measured leaf reflectance and chlorophyll a fluorescence and performed phytohormone assays following inoculation with *Blumeria graminis* f. sp. *hordei*. The study focused on a susceptible wild-type barley cultivar and its near-isogenic lines carrying various resistance genes: *Mla*, *Mlg* and *mlo*. The authors showed that the resistant lines (*mlo* and *Mlg*) exhibited a high activity of the photosynthetic apparatus, which was associated with higher energy conservation during photosynthesis that may be a part of protective mechanisms during powdery mildew inoculation. Furthermore, the resistant lines *Mlg* and *Mla* were characterized by different levels of growth and stress hormones.

The aim of a study by Wójcik-Jagła et al. [6] was to select candidate genes involved in responses to freezing (six genes, encoding elongation factor 1 alpha (EF1A), ferredoxin-NADP reductase, a 14-3-3a protein, β-fructofuranosidase, CBF2A and CBF4B) and drought (six genes, encoding transketolase, periplasmic serine protease, triosephosphate isomerase, a protein with a cochaperone region (*GroEs*), pfam14200 and actin). Their expression in barley under both stress factors was also analyzed. The authors confirmed the role of the candidate genes in the tolerance to freezing and drought, observed in the population of winter barley DH (doubled haploid) lines.

Sadura et al. [7] undertook a study to answer the question of how a deficiency in brassinosteroids (BR) or disruption in their signaling may induce the accumulation of heat-shock proteins (HSP90, HSP70, HSP18 and HSP17), both in the form of transcripts and of proteins themselves, in barley exposed to low and high temperatures. Hence, the BR-deficient (mutations in *HvDWARF* or *HvCPD*) and BR-signaling (mutation in *HvBRI1*) mutants were used in the experiment. The BR-signaling mutant was characterized by decreased levels of the HSP transcripts and heat-shock proteins. In the BR-deficient mutants, the reduced accumulation of HSP70 and HSP90 transcripts was associated with increased accumulation of these HSPs. The authors discussed their results in terms of altered plant tolerance to more extreme temperatures.

The article by Ube et al. [10] focused on the identification of 9-hydroxy-8-oxotryptamine, N-cinnamoyl-8-oxotryptamine and N-cinnamoyl-(1H-indol-3-yl)methylamine in *Fusarium*-infected barley. The above compounds are classified as triticamides A–C and characterized as barley phytoalexins. Additionally, nine acyltransferase genes (*HvTHT1*—*HvTHT9*), encoding enzymes that catalyze phenylamide synthesis, were also characterized. The results proved that HvTHT7 and HvTHT8 contribute to the biosynthesis of phenylamide phytoalexins in pathogen-infected barley. 

## 4. Reviews 

The review by Gietler et al. [11] presents two faces of abscisic acid (ABA): a savior and a fiend. Its positive role involves its contribution to the control of plant natural developmental processes, as well as to stress responses. In wheat and barley, the role of ABA is well recognized in the majority of abiotic stresses. However, changes in ABA content also affect cereal sensitivity to adverse environmental factors. This review presents examples demonstrating that the role of ABA is not always associated with survival mechanisms. In certain cases, ABA may be a factor increasing plant susceptibility to biotic and abiotic stresses. The authors concluded that ABA involvement in response to biotic stresses is not yet thoroughly understood, and that this problem seems to be of importance for determining future directions in cereal breeding programs.

Kong et al. [12] provide an overview of recent studies concerning epigenetic processes and elements (DNA methylation, histone modification, chromatin remodeling, noncoding RNAs) involved in plant responses to environmental stresses in wheat and barley. Additionally, a general model for the role of epigenetic elements and processes in stress responses in wheat and barley is proposed. The authors discuss the potential of exploiting epigenetic variations in the search for improvement of agricultural traits in both cereals.

## 5. Conclusions

This Special Issue is a summary of the latest research on the mechanisms of wheat and barley acclimatization to environmental stresses. Several genes and mechanisms were indicated, which can be used in precision breeding to improve the tolerance of these two cereal species to individual stress factors. The experimental outcomes form a starting point for further studies and new mechanistic research hypotheses, as emphasized in the majority of the referenced works. This Special Issue will provide its readers with several guidelines, molecular tools and strategies that can be used in similar studies focusing on improving tolerance in cereals and other crops. I hope that the reports published in this Special Issue will contribute to better understanding of the molecular mechanisms underlying wheat and barley acclimatization to environmental stresses, while they will set new directions for further research, to fill the existing gaps and find at least partial answers to still-unanswered questions.

I also remain most grateful to the authors of these publications for sharing their findings with a wide audience including plant biologists and wheat and barley breeders. 

## Figures and Tables

**Table 1:** Contributors to the Special Issue “Wheat and Barley: Acclimatization to Abiotic and Biotic Stress”.

Authors	Keywords
Zhang et al. [1]	Heavy metal-associated (HMA) isoprenylated plant proteins (HIPPs); gene family; *Haynaldia villosa* L.; subcellular localization; Cd (cadmium) tolerance
Liu et al. [2]	wheat powdery mildew; *Haynaldia villosa*; *CMPG1-V*; RNA-seq; hormone signaling; metabolism process
Saja et al. [3]	barley; *Blumeria graminis*; chlorophyll a fluorescence; leaf reflectance; phytohormones; resistance genes
Terletskaya et al. [4]	alloplasmic wheat lines; drought tolerance; photosynthesis; *DREB*; *orf256*; *rps19-p* genes; SSR (simple sequence repeat)
Zhi et al. [5]	HDT701; HDA6; histone modification; wheat; *Blumeria graminis* f.sp. *tritici*
Wójcik-Jagła et al. [6]	barley; doubled haploid lines; freezing tolerance; drought tolerance; candidate genes; gene expression
Sadura et al. [7]	brassinosteroids; acclimation process; small heat-shock proteins (sHSPs); HSP70; HSP90; temperature stress
Ye et al. [8]	*Bipolaris sorokiniana*; comparative transcriptome; pathogenicity; cell wall-degrading enzymes; toxin producing; defensive response
Czyczyło-Mysza et al. [9]	doubled haploid lines; epistasis; genetic map; water deprivation stress; *Triticum aestivum*
Ube et al. [10]	*Hordeum vulgare*; phytoalexin; phenylamide; triticamide; tryptamine N-hydroxycinnamoyl transferase; *Bipolaris sorokiniana*; *Fusarium culmorum*; *Fusarium graminearum*
Gietler et al. [11]	abiotic stresses; abscisic acid (ABA); barley (*Hordeum vulgare* L.); biotic stresses; wheat (*Triticum aestivum* L.)
Kong et al. [12]	epigenetic; abiotic and biotic stress; wheat; barley; DNA methylation; histone modification; chromatin remodeling; noncoding RNAs
